# Metabolomics, a Powerful Tool for Agricultural Research

**DOI:** 10.3390/ijms17111871

**Published:** 2016-11-17

**Authors:** He Tian, Sin Man Lam, Guanghou Shui

**Affiliations:** State Key Laboratory of Molecular Developmental Biology, Institute of Genetics and Developmental Biology, Chinese Academy of Sciences, Beijing 100101, China; tianhe@genetics.ac.cn (H.T.); smlam@genetics.ac.cn (S.M.L.)

**Keywords:** metabolomics, lipidomics, *Arabidopsis*, stresses, NMR, MS

## Abstract

Metabolomics, which is based mainly on nuclear magnetic resonance (NMR), gas-chromatography (GC) or liquid-chromatography (LC) coupled to mass spectrometry (MS) analytical technologies to systematically acquire the qualitative and quantitative information of low-molecular-mass endogenous metabolites, provides a direct snapshot of the physiological condition in biological samples. As complements to transcriptomics and proteomics, it has played pivotal roles in agricultural and food science research. In this review, we discuss the capacities of NMR, GC/LC-MS in the acquisition of plant metabolome, and address the potential promise and diverse applications of metabolomics, particularly lipidomics, to investigate the responses of *Arabidopsis thaliana*, a primary plant model for agricultural research, to environmental stressors including heat, freezing, drought, and salinity.

## 1. Introduction

Metabolomics, defined as a powerful platform for the global identification and quantification of low-molecular-weight metabolites in a biological sample, is rapidly evolving into a principal tool in the functional annotation of genes; and confers high-resolution snapshot of various physiological and biological aspects of cellular responses to specific environmental stimuli [1].

The field of metabolomics relies mainly on the technologies of nuclear magnetic resonance (NMR) and mass spectrometry (MS) with or without chromatography, which allows the detection of an organism’s metabolite pool characterized by plethora of chemical structures with an enormous diversity of chemical and physical properties [1,2,3,4,5]. Since its introduction by Nicholson et al. in 1999, metabolomics has been extensively applied to various fields of science in the post-genomic era, such as agricultural research [6,7,8,9,10].

*Arabidopsis thaliana* was one of the first established model organisms worldwide [11], and has been extensively studied, rendering it an ideal research model. Metabolomics has been broadly applied to a myriad of studies pertaining to the metabolic responses of *Arabidopsis* to various abiotic or biotic stresses, including heat, freezing, drought, and salinity [12,13]. Lipidomics, as a comparably young branch in the realm of metabolomics area, has emerged as a burgeoning arena within the last few years [14]; and has been applied to various analyses to elucidate the physiological mechanisms of *Arabidopsis* [15,16,17,18,19,20].

**Figure 1:**
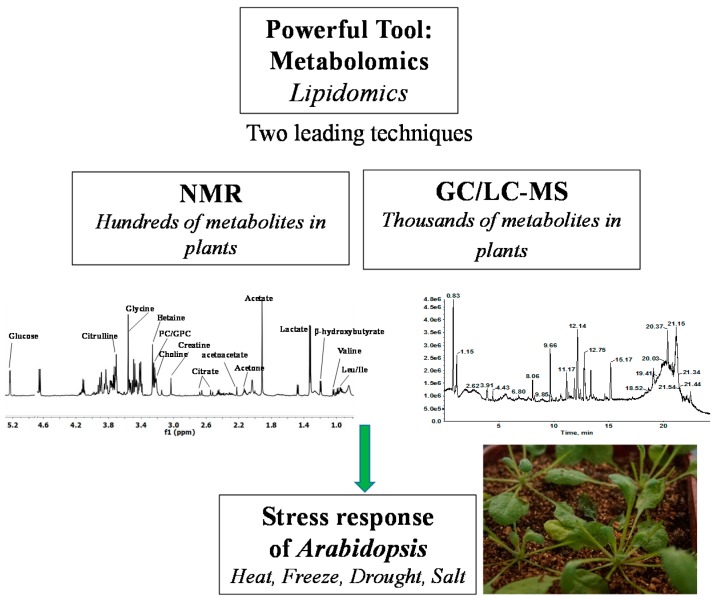
Two leading techniques, nuclear magnetic resonance (NMR) and gas chromatography/liquid chromatography–mass spectrometry (GC/LC-MS), and their applications to the elucidation of the physiological mechanisms of *Arabidopsis* to various environmental stressors.

In this review, we discuss the capacity of NMR and gas-chromatography/liquid-chromatography coupled to mass spectrometry (GC/LC-MS) in acquiring qualitative and quantitative information on carbohydrates, amino acids, organic acids, and lipids in plants, as well as the application of metabolomics and lipidomics in providing the mechanistic details of the physiological responses of *Arabidopsis* to heat, freezing, drought, and salt stresses. The technical routes of this review are illustrated in Figure 1.

## 2. Techniques of Plant Metabolome Acquisition

Quantitative plant metabolomics provides us with an in-depth understanding of plant metabolism, and helps to improve crop yields [21]. At present, NMR and GC/LC-MS techniques dominate the data acquisition strategies in metabolomics studies, which enable the identification of a myriad of species belonging to the three major classes of nutrient components (i.e., carbohydrate, amino acid, and lipids) in plants [6,21]. Other chromatographic techniques, including capillary electrophoresis (CE) and supercritical fluid chromatography (SFC), can also be coupled to MS for metabolomics studies. However, CE and SFC are being applied to a less extensive extent than conventional GC/LC analyses due to the drawback of poor migration time reproducibility and lack of reference libraries for CE [22], and fluid compressibility for SFC [23].

### 2.1. Metabolite Coverage of NMR in Plant Metabolomics

NMR spectroscopy, one of the two leading analytical techniques in the field of metabolome research, is characterized by its reproducibility in quantification, structure identification, and non-biased detection of metabolites [24]. NMR can quantify metabolites in large batches of samples with higher reproducibility and greater accuracy, coupled with a wider time span and dynamic ranges than GC/LC-MS can perform. Particularly in untargeted MS-based metabolomics, the measurements are semi-quantitative. NMR guarantees stable sensitivity as samples and instruments are devoid of contact during detection, eliminating the concerns of gradual contamination by residual metabolites that may compromise sensitivity in MS analyses. In addition, NMR provides the same signal sensitivity for all metabolites regardless of the complexities of the biological matrix, and is independent of the chemical properties of the metabolites [25]. NMR is a powerful technique for the analysis of metabolite structures, as it can differentiate compounds with identical masses and two-dimensional structures that differ only in spatial configuration [26,27]. NMR is also preferred in metabolomics studies due to its simple detection requirements using intact bio-specimens without requirements for prior separation.

In terms of plant metabolome analysis, NMR mainly covers metabolites, carbohydrate, amino acids, and organic acids [6,7,8].

A major drawback of NMR technique, however, lies in its sensitivity [24,25,28], which restrains its application to the detection of metabolites of low-abundance in plants. In addition, as molecular weight increases, for example, for lipids comprising long fatty chains, its identification capacity is weakened rapidly due to more complex and overlapping signals from the extended hydrocarbon chains in such compounds. The long-carbon-chain lipids, such as fatty acids (FAs) and phospholipids carrying single or multiple long fatty chains, can be further differentiated into hundreds of thousands of subtype species according to the lengths of fatty chains, the number of double bonds, and the functional groups, etc. As NMR can only provide classification based on the characteristic signals located in functional groups, it falls short in terms of conferring specific compound identification due to overlapping signals of methylene in long fatty chains. As can be seen from Table 1, only hundreds of metabolites can be identified by NMR, which falls way behind GC/LC-MS.

### 2.2. Metabolite Coverage of GC/LC-MS in Plant Metabolomics

MS coupled to GC or LC is by far the most frequently applied analytical technique in plant metabolomics studies due to its unparalleled sensitivity and extensive coverage of biological information relevant to the metabolism of the organism [29,30]. The plant metabolome reported to date is composed of approximately 30,000 endogenous metabolites that mainly comprises various lipid classes, the majority of which can be easily characterized and quantified via MS.

GC-MS is excellent for the detection of biological samples with very complex matrices, offering highly efficient separation and resolution. Not only can it analyze many of the aforementioned carbohydrates, amino acids, and organic acids detectable by NMR, but it aslo accurately identify a plethora of volatile and thermally stable lipids, or volatile derivatized metabolites, such as FAs [9,31,32]. More importantly, an immense number of well-curated compound reference libraries, including the NIST [33], FiehnLib [34] and Golm metabolic databases (GMD [35]) are availabe for peak identification and prediction across different models of mass spectrometers, which have been proven very useful in terms of analysing metabolome data. Major limitations of GC-MS, however, lie in its inability to ionize thermolabile metabolites, such as di- and triphosphates, lysophosphatidycholine (LPC), lysophosphatidylethanolamine (LPE), or higher molecular masses phosphatidylcholine (PC) and phosphatidylethanolamine (PE), due to their non-volatile properties even after derivatization, circumscribing its application for global metabolic profiling in plants. This limitation narrows the GC-MS-derived metabolome both in terms of metabolite number and subtypes compared to that obtained using LC-MS (Table 1).

In comparison to GC-MS, LC-MS is useful in handing thermolabile, polar metabolites, and high-molecular weight compounds without derivatization, such as phosphatidylinositol (PI), PE, phosphatidic acid (PA), phosphatidylglycerol (PG), phosphatidylserine (PS), sulfoquinovosyldiacylglycerol (SQDG), PC, monogalactosyldiacylglycerol (MGDG), and digalactosyldiacylglycerol (DGDG) [15,16,36]. More importantly, with the advancement in ionization techniques, increasing scan speed, and improvement in terms of instrument sensitivity, metabolite coverage of LC-MS can be expanded into greater array of metabolite classes, traditionally dominated by GC-MS [37,38,39]. For example, volatile metabolites involved in tricarboxylic acid cycle (TCA), which are generally detected by GC-MS, can now also be analyzed by LC-MS [40]. Even though GC-MS exhibits higher senstitivity for these volatile compounds aforementioned than LC-MS; the detectability of such compounds in LC-MS per se is enough for the quantitative analysis of targeted metabolites in plant.

## 3. The Application of Metabolomics to *Arabidopsis* Model-Based Research

Metabolomics has been widely applied to a number of studies pertaining to the metabolic responses of *Arabidopsis* to various abiotic or biotic stresses. In this part, we summarized the potential promise and diverse applications of metabolomics, particularly lipidomics, to investigate the responses of *Arabidopsis* to environmental stressors including heat, freezing, drought and salinity (Table 2).

### 3.1. Temperature Stress-Induced Alterations of Arabidopsis Metabolome

Environmental stressors, including heat, freezing, drought, and salinity, are detrimental to the normal growth of plants [51,52,53]. The sessile nature of plants makes them particularly susceptible to such environmental stressors, and thus plants have evolved various physiological and metabolic responses to effectively deal with such environmental extremities [54]. Temperature extremes can lead to oxidative stress, which is extremely harmful to various biomolecules including lipids, nucleic acids, and proteins in plants [55,56].

Temperature extremes can lead to severe crop-yield losses [52,53,57,58], and elevated temperature is predicted to result in severe food crises in the future with global warming on the rise [59,60]. To improve thermotolerance in crops, it is essential to understand the molecular basis of thermotolerance adaptation in plants.

On the other hand, low temperature represents a key determinant in influencing the geographical distribution of plants worldwide. Cold acclimation is an important mechanism that enables plant species to survive through the low temperature of the harsh winters in their natural habitats [61]. Complex changes in plant transcriptome, proteome, and metabolome occurs during the cold acclimation process [61,62], initiating specific mechanisms to protect plants against freezing [63].

Cold stress (CS) influenced plant metabolism far more profoundly than heat stress (HS). Analysis on temperature-stressed metabolome of *Arabidopsis* revealed that, with regard to metabolite markers, heat stress and cold stress shared many comparable responses [13]. Increased concentrations of branched-chain amino acids (BCAA), like isoleucine (Ile), leucine (Leu), and valine (Val), and aromatic amino acids (AAA), such as tyrosine (Tyr) during both temperature stresses were detected. Increased concentrations of tryptophan (Trp) and phenylalanine (Phe) were, however, only observed during cold accumulation [8].

Salicylic acid, which plays a crucial function in guarding the plant hosts against pathogen intrusion [13,64,65,66,67,68], was enhanced during both HS and CS accumulations, suggesting that salicylic acid could be the initial signaling molecule of plant tolerance to various stresses, and could possibly help plants prepare for combat against pathogens.

The promoters of a number of genes induced by temperature stresses contain sugar-responsive elements [13,69,70], indicating that sugar signaling may be important in the establishment and maintenance of both acquired heat and freeze tolerance. A metabolic profiling study on *Arabidopsis* showed that gluconapin is characteristic in CS accessions, while kaempferol-3,7-*O*-dirhamnoside and kaempferol-neohesperidoside-7-rhamnoside are specific to highly cold-tolerant accessions, which can facilitate the screening of cold tolerance in *Arabidopsis* accessions [71].

Secondary metabolism-generated metabolites, such as Ile, Leu, Val, Tyr, Trp, and Phe can defend against pests, pathogenic fungi, and bacteria [72]. It is plausible that BCAA accumulation functions to enhance secondary metabolism, facilitating the development of resistance against pathogens during stress conditions.

In plants, cold response and day-night cycles share many regulation genes [73]. Metabolic and transcriptional analyses on *Arabidopsis* exposed to CS revealed that the expression of day-regulation genes was profoundly influenced by CS, indicating a disrupted clock function, and the importance of understanding the mechanism of cold acclimation in the correct day-night context.

Environmental conditions, especially temperatures, can directly affect cell membrane properties, which subsequently influence membrane fluidity, and this can be balanced by plants via regulation of membrane glycerolipids saturation indices, as the presence of unsaturated bonds decreases the phase-transition temperature [74]. In this area, lipidomics has been broadly applied to the study of the influence of temperature on *Arabidopsis* [36]. In the initial stage of adaptation to HS (32 °C), increases in PG 32:0 and SQDG 36:5 were observed in *Arabidopsis*, while PE 36:6, PG 36:4, and PG 36:5 showed decreases in response to HS, indicating that membrane lipid compositions can be fine-tuned to counter temperature changes, principally via modulating desaturation leading to compensatory decreases in membrane fluidity in response to high temperatures. Furthermore, SGDG, PG, and PE represent anionic glycerolipids reported to aid in stabilizing membrane proteins [75].

With the advances in analytical methods in the field of lipidomics, a LC-MS lipidomic platform was developed, which extended the coverage of plant lipidome [43]. In particular, PG (16:0/18:3) concentration was up-regulated upon cold acclimation. In addition, the degree of unsaturation in long-chain bases of sphingolipids was observed to increase during CS [43], presumably enhancing *Arabidopsis* CS tolerance [76].

Moreover, lipid changes were reported during all phases of the freezing/cold exposure, including cold acclimation, the freezing process per se, as well as recovery from low temperature [77,78,79,80]. CS-induced lipid alterations can limit detrimental lipid phase changes that may result in the cell membrane leakage [77]. Indeed, CS- or freezing-induced alterations in plant lipid metabolism can modulate subsequent damage during stress exposure [20,77,78,79]. For instance, desaturases producing trienoic fatty acids are required for effective photosynthesis under cold conditions [80]. Therefore, much still remains to be explored on the role of lipids in cold and freezing responses, and these necessitate the development of more sensitive and extensive lipidomics and metabolomics analytical strategies tailored to *Arabidopsis*.

### 3.2. Drought-Stress (DS) Induced Alterations of Arabidopsis Metabolome

DS can result in significant loss of plant productivity [81,82,83]. In the drought of 2012 in the United States, the most severe drought experienced in the past 25 years, the production and yields of corn and soybean were severely afflicted, leading to huge economic repercussions [84]. Such weather extremities call for a better understanding of the metabolic mechanisms of stress response, which would be crucial for improving crop tolerance to ensure agricultural outputs and economic stability [85].

Flavonoids represent a major component of specialized/secondary metabolites in plants [86] that are thought to be defense metabolites against environmental stresses [87], and accumulation of flavonoids in *Arabidopsis* have been previously observed in response to various stresses [88,89,90,91,92,93,94]. It was reported that flavonoid accumulation, which was shown to participate in radical scavenging activity, increases the oxidative and drought tolerance of plants, thereby preventing water loss [95].

Increased concentrations of three types of flavonoids, glycosides of kaempferol (f1, f2, and f3), quercetin (f6 and f8), and cyanidin (A5, A8, A9, A10, and A11) were noted in *Arabidopsis* during DS, indicating that all flavonoids are DS responsive metabolites that can be used as positive markers and potential mitigators of DS [44]. Nonetheless the signaling/regulation mechanisms of flavonoids or the individual role of each molecule in the stress mitigation mechanism has remained unclear.

The diversity of secondary metabolites is critical for their roles in plant stress response [96]. Metabolomics can aid transcriptomics to elucidate cellular machinery of metabolic tolerance towards stress. The characterization of the flavonoid pathway transcription factor TRANSPARENT TESTA8 (TT8) in *Arabidopsis* using an integrative omics strategy revealed that two phytohormone biosynthesis pathways of jasmonic acid and brassinosteroids, which are implicated in stress response [96], are directly regulated by TT8 [97]. Furthermore, at least eight stress response proteins, which are implicated in the tolerance against salt and drought stress [98,99,100], are directly regulated in a TT8-dependent manner, thus implicating TT8 in reprogramming defense response. In addition, TT8 has a direct role in increasing the diversity of core metabolites, particularly by regulating glycosylation of brassinosteroids and flavonoids.

Mitochondrial metabolism was reported to be highly active during DS responses [45]. DS-induced metabolic reprogramming leads to up-regulated concentrations of amino acids and intermediates from TCA cycle, including cis-aconitate, isocitrate, citrate, fumarate, 2-oxoglutarate, succinate, malate, glycolate, putrescine, spermidine, gamma-aminobutyric acid (GABA,) guanidine, fructose, galactose, glucose, maltose, mannose, raffinose, ribose, sucrose, trehalose, dehydroascorbate, alanine (Ala), aspartate (Asp), glutamate (Glt), glutamine (Gln), Ile, Leu, lysine (Lys), methionine (Met), ornithine (Orn), Phe, proline (Pro), serine (Ser), threonine (Thr), Try, and Val, as well as the decreased concentrations of protein, starch and nitrate. The increased concentrations of the BCAA appear to be associated with their increased utilization as TCA cycle substrates [101,102], and functions in the short-term DS, most likely by retarding stress initiation. A multiplicity of primary metabolites (osmolytes, osmoprotectants) accumulate under stress conditions, which could serve as building blocks for macromolecules to stabilize membranes and therefore contribute to cell osmotic pressure [9,13]. Indeed, this may be why it is often infeasible to generate powerful stress tolerance varieties via engineering the overproduction of merely a single compatible solute compound in plants.

Lipids, as major membrane components, function to preserve the membrane integrity during DS process [47]. Concentrations of glycosylinositolphosphoceramide (GIPC), steryl glycoside (SG), acylated steryl glycoside (ASG), DGDG, and PA in *Arabidopsis* were reported to be altered by DS [43,47,48]. In response to DS, increased DGDG 18:3 and PC 18:3, and diminished levels of triunsaturated fatty acid 16:3 and 18:3 were observed in leaves of *Arabidopsis* [47]. These results indicated that *Arabidopsis* have a strong capacity for tolerating DS at the cellular level via modulating membrane lipid proportions.

### 3.3. Salt Stress-Induced Alterations in Arabidopsis Metabolome

Soil and water salinity is pervasive throughout the globe, and may adversely impact the crop yield [103]. Plants have evolved tolerance against saline stress, which may involve osmotic adjustment and the sequestration of ions into respective cellular compartments [104]. Numerous metabolomic studies that elucidate the tolerance mechanisms of *Arabidopsis* in response to salt stress have been reported [105,106].

Using global transcriptional and metabolomic analyses, a study on the regulation mechanism of polyamine in *Arabidopsis* salinity tolerance revealed that activation of abscisic acid and jasmonate biosynthesis, and accumulation of important compatible solutes; as well as TCA cycle intermediates, was observed [49]. Expression analyses indicated that thermospermine regulates the transcript levels of certain target genes associated with the biosynthesis and signaling of jasmonate, some of which are proven to enhance salinity tolerance.

The osmotic potential in the plants cell is increased upon hyperosmolarity challenge. The osmotic potential of the cell cytosol is reduced by plant cells via building up compatible osmolytes to maintain the activity of enzymes [46]. Concentrations of Pro, histidine (His), glutathione, and GABA in *Arabidopsis* were strongly affected by salt stress. Pro, polyamines, organic acids, and Gly betaine, as compatible osmolytes, can greatly decrease stress-induced destruction of plant cells [107,108,109,110,111,112]. The compatible osmolytes are synthesized via shifting basic intermediary metabolites towards stress-activated biochemical reactions. Cytochrome P450s generally participate in primary and secondary metabolism and may be implicated in some osmolytes biosynthesis. Many other potential biochemical compounds, which are associated with salt tolerance, can also be uncovered by metabolic analysis [113,114,115].

## 4. Future Perspectives

Although metabolomics represents a relatively new realm of science that emerged not more than two decades ago, it has already exhibited considerable potential in agricultural and food science research, especially pertaining to the elucidation of metabolome and lipidome changes in response to environmental or pathophysiological stimuli; thereby contributing to the crop production improvement. Despite the rapid advances in the techniques of NMR and MS coupled to GC or LC, and their broad applications to the field of metabolomics, thus far, no single analytical platform by itself can offer a holistic coverage of the metabolome in plants. While NMR possesses the advantage of capturing the structural information of metabolites as well as their abundances, LC-MS emerges as the probable optimal choice in terms of the acquisition of global metabolome in plants, and the rapid advances in MS instrumentation is greatly driving its diverse applications in agricultural sciences.

In face of adverse environmental conditions, a series of primary metabolites (osmolytes, osmoprotectants) and secondary metabolites (defense metabolites) in plants accumulate to enhance their stress tolerance, thus it is impossible to generate plants with high levels of stress adaptation via engineering the levels of a just a few selected metabolites. Future studies will focus on increasing the resolution and coverage of the metabolome to obtain a comprehensive understanding of how plants adapt themselves to environmental stresses, providing new avenues to increase crop production.

Apart from the identification of critical metabolic pathways to uplift crop production, metabolomics also confers a powerful tool to assess food crop quality. For example, global profiling of plant/crop metabolome could also facilitate in the identification of genetic manipulations that may serve to increase the nutritional value and quality of crops. For instance, previous lipidomic analysis has revealed that GmMYB73 manipulation promotes lipid accumulation in soybeans, thus providing a potential avenue for increasing oil production in legume crop plants [116]. Another example is given by the establishment of an integrated lipidomic approach comprising multiple analytical arms specifically tailored to the analysis of the fine lipidomic fingerprints of palm oil, currently the leading edible oil consumed around the globe, which could be applied to the evaluation of oil quality and the health benefits/risks associated with different oil refinement techniques [117]. Again, resolution of the metabolome (lipidome) is instrumental in such applications, since a highly sensitive analytical methodology is indispensable in detecting trace amounts of critical components such as oxidized lipids including oxidized triacylglycerols that could be auxiliary in determining the oil quality.

In conclusion, while metabolomics holds great promise in forwarding agricultural research, a pressing need exists to expand its analytical capacity in order to derive fully integrated functional networks of metabolites that have true biological meaning. The systemic construction of metabolome libraries with sufficient resolution is therefore expected to further broaden the translational applications of metabolomics in various sub-arenas of agricultural research. Finally, the plethora of metabolomics data also calls for the development of competent information processing tools that allow data to be processed, integrated, interpreted, and verified alongside with proteomics and transcriptomics strategies in order to fully unravel the various intricate biological networks under study.

## Figures and Tables

**Table 1:** Coverages of plant metabolome by NMR and GC/LC-MS techniques.

Techniques	Metabolite Coverage	Advantages and Disadvantages
NMR	Hundreds of metabolites	Same signal sensitivity for all metabolites, independent of metabolite properties and matrix effects, powerful ability in structural elucidation of plant metabolomics [24,25,26,27,30]
	Mainly carbohydrates, organic acids, amino acids, etc. [6,7,8,25]	Less sensitive than MS [24,25,28,30]
GC-MS	Thousands of metabolites	Sensitive for the detection of volatile, thermally stable metabolites [31,32]
	Mainly carbohydrates, organic acids, amino acids, fatty acids (Fas), etc. [25,31,32]	Inability for the detection of thermolabile metabolites [30,41]
LC-MS	Thousands of metabolites	Sensitive for thermoliable, polar metabolites, and high-molecular weight metabolites [30,41]
	Mainly carbohydrates, organic acids, amino acids, lipids (PI, PE, PA, PG, PS, SQDG, PC, MGDG, DGDG etc.), etc. [15,25,36]	Weak sensitivity for samples with high salt content, less sensitive for the detection of volatile metabolites [31,32]

PI: phosphatidylinositol; PE: phosphatidylethanolamine; PA: phosphatidic acid; PG: phosphatidylglycerol; PS: phosphatidylserine; SQDG: sulfoquinovosyldiacylglycerol; MGDG: monogalactosyldiacylglycerol; and DGDG: digalactosyldiacylglycerol.

**Table 2:** Metabolomic profiling of the physiological responses to heat, freezing, drought, and salt stresses in *Arabidopsis*.

Stresses	Metabolic Markers	Alterations	Pathway	References
Heat	PG 32:0, SQDG 36:5, TG 54:6, TG 54:7, TG 54:8, TG 54:9	↑	Lipid	[36,42]
	PE 36:6, PG 36:4, PG 36:5	↓		
	GABA, AA	↑	Amino acid	[13]
	Maltose, raffinose, trehalose	↑	Amino sugar metabolism	[13]
	Putrescine, glycerol, salicylic acid, malate, succinate	↑	Carbohydrate	[13]
Freeze	Maltose, raffinose, trehalose	↑	Amino sugar metabolism	[13]
	Malate, succinate, salicylic acid, gluconapin, putrescine, glycerol, kaempferol-3,7-*O*-dirhamnoside, kaempferol-neohesperidoside-7-rhamnoside	↑	Carbohydrate	[13]
	GABA, BCAA, AAA	↑	Amino acid	[13]
	PG (16:0/18:3)	↑	Lipid	[43]
	PG (16:1/18:2), PG (16:1/18:3)	↓		
Drought	Anthocyanin, flavonoid, glycosides, *cis*-aconitate, isocitrate, putrescine, spermidine, guanidine, dehydroascorbate, citrate, fumarate, 2-oxoglutarate, succinate, malate, glycolate	↑	Carbohydrate	[44,45]
	Fructose, galactose, glucose, maltose, mannose, raffinose, ribose, sucrose, trehalose	↑	Amino sugar metabolism	[45]
	GABA, BCAA, AAA	↑	Amino acid	[46]
	DGDG 18:3, PC 18:3, TG 54:6, TG 54:7, TG 54:8, TG 54:9	↑	Lipid	[42,47]
	Triunsaturated FA 16:3 and 18:3	↓	Lipid	[45]
	GIPC, ASG, SG	↑	Lipid	[43]
	Phosphatidic acid	↓	Lipid	[48]
Salt	GABA, BCAA, AAA	↑	Amino acid	[46,49]
	Jasmonate	↑	Lipid	[49]
	Disaccharides, sucrose, fructose, raffinose, myo-inositol	↑	Amino sugar metabolism	[50]
	malate, citrate, 2-ketoglutarate, succinate	↑	TCA	[50]
	ethanolamine, valine, leucine, proline, glycine	↑	Amino acid	[50]
	TG 54:6, TG 54:7, TG 54:8, TG 54:9	↑	Lipid	[42]

“↑” and “↓”: represents up- and down-regulated concentration in response to stress, respectively; GABA: gamma-aminobutyric acid; AA: amino acid; BCAA: branched-chain amino acid; AAA: aromatic amino acid; GIPC: glycosylinositolphosphoceramide; ASG: acylated steryl glycoside; SG: steryl glycoside.

## Abbreviations

Ile	Isoleucine
Val	Valine
Trp	Tryptophan
Ala	Alanine
Glt	Glutamate
Lys	Lysine
Orn	Ornithine
Ser	Serine
His	Histidine
Leu	Leucine
Tyr	Tyrosine
Phe	Phenylalanine
Asp	Aspartate
Gln	Glutamine
Met	Methionine
Pro	Proline
Thr	Threonine
Gly	Glycine
AA	Amino acid
AAA	Aromatic amino acid
LPC	Lysophosphatidycholine
PC	Phosphatidylcholine
PI	Phosphatidylinositol
PG	Phosphatidylglycerol
SQDG	Sulfoquinovosyldiacylglycerol
DGDG	Digalactosyl-diacylglycerol
GABA	Gamma-Aminobutyric acid
SG	Steryl glycosides
BCAA	Branched-chain amino acid
FA	Fatty acid
LPE	Lysophosphatidylethanolamine
PE	Phosphatidylethanolamine
PA	Phosphatidic acid
PS	Phosphatidylserine
MGDG	Monogalactosyldiacylglycerol
TCA	Tricarboxylic acid cycle
GIPC	Glycosylinositolphosphoceramides
ASG	Acylated steryl glycosides

## References

[1] Saito K., Matsuda F. (2010). Metabolomics for functional genomics, systems biology, and biotechnology. Annu. Rev. Plant Biol..

[2] Simó C., Ibáñez C., Valdés A., Cifuentes A., García-Cañas V. (2014). Metabolomics of genetically modified crops. Int. J. Mol. Sci..

[3] Tebani A., Abily-Donval L., Afonso C., Marret S., Bekri S. (2016). Clinical Metabolomics: The New Metabolic Window for Inborn Errors of Metabolism Investigations in the Post-Genomic Era. Int. J. Mol. Sci..

[4] Washio J., Takahashi N. (2016). Metabolomic Studies of Oral Biofilm, Oral Cancer, and Beyond. Int. J. Mol. Sci..

[5] Hong J., Yang L., Zhang D., Shi J. (2016). Plant Metabolomics: An Indispensable System Biology Tool for Plant Science. Int. J. Mol. Sci..

[6] Hagel J.M., Mandal R., Han B., Han J., Dinsmore D.R., Borchers C.H., Wishart D.S., Facchini P.J. (2015). Metabolome analysis of 20 taxonomically related benzylisoquinoline alkaloid-producing plants. BMC Plant Biol..

[7] Song E.H., Kim H.J., Jeong J., Chung H.J., Kim H.Y., Bang E., Hong Y.S. (2016). A ^1^H HR-MAS NMR-Based Metabolomic Study for Metabolic Characterization of Rice Grain from Various *Oryza sativa* L. Cultivars. J. Agric. Food Chem..

[8] Tomita S., Ikeda S., Tsuda S., Someya N., Asano K., Kikuchi J., Chikayama E., Ono H., Sekiyama Y. (2016). A survey of metabolic changes in potato leaves by NMR-based metabolic profiling in relation to resistance to late blight disease under field conditions. Magn. Reson. Chem..

[9] Angelcheva L., Mishra Y., Antti H., Kjellsen T.D., Funk C., Strimbeck R.G., Schröder W.P. (2014). Metabolomic analysis of extreme freezing tolerance in *Siberian spruce* (*Picea obovata*). New Phytol..

[10] Misra B.B., Yin Z., Geng S., de Armas E., Chen S. (2016). Metabolomic Responses of *Arabidopsis* Suspension Cells to Bicarbonate under Light and Dark Conditions. Sci. Rep..

[11] Wienkoop S., Baginsky S., Weckwerth W. (2010). *Arabidopsis thaliana* as a model organism for plant proteome research. J. Proteom..

[12] Qi X., Zhang D. (2014). Plant metabolomics and metabolic biology. J. Integr. Plant Biol..

[13] Kaplan F., Kopka J., Haskell D.W., Zhao W., Schiller K.C., Gatzke N., Sung D.Y., Guy C.L. (2004). Exploring the temperature-stress metabolome of *Arabidopsis*. Plant Physiol..

[14] Harkewicz R., Dennis E.A. (2011). Applications of mass spectrometry to lipids and membranes. Annu. Rev. Biochem..

[15] Nakamura Y., Teo N.Z., Shui G.H., Chua C.H., Cheong W.F., Parameswaran S., Koizumi R., Ohta H., Wenk M.R., Ito T. (2014). Transcriptomic and lipidomic profiles of glycerolipids during *Arabidopsis* flower development. New Phytol..

[16] Nakamura Y., Koizumi R., Shui G.H., Shimojima M., Wenk M.R., Ito T., Ohta H. (2009). *Arabidopsis* lipins mediate eukaryotic pathway of lipid metabolism and cope critically with phosphate starvation. Proc. Natl. Acad. Sci. USA.

[17] Li M., Baughman E., Roth M.R., Han X., Welti R., Wang X. (2014). Quantitative profiling and pattern analysis of triacylglycerol species in *Arabidopsis* seeds by electrospray ionization mass spectrometry. Plant J..

[18] Vu H.S., Shiva S., Hall A.S., Welti R. (2014). A lipidomic approach to identify cold-induced changes in *Arabidopsis* membrane lipid composition. Methods Mol. Biol..

[19] Okazaki Y., Kamide Y., Hirai M.Y., Saito K. (2013). Plant lipidomics based on hydrophilic interaction chromatography coupled to ion trap time-of-flight mass spectrometry. Metabolomics.

[20] Degenkolbe T., Giavalisco P., Zuther E., Seiwert B., Hincha D.K., Willmitzer L. (2012). Differential remodeling of the lipidome during cold acclimation in natural accessions of *Arabidopsis thaliana*. Plant J..

[21] Fernandez O., Urrutia M., Bernillon S., Giauffret C., Tardieu F., Le Gouis J., Langlade N., Charcosset A., Moing A., Gibon Y. (2016). Fortune telling: Metabolic markers of plant performance. Metabolomics.

[22] Obata T., Fernie A.R. (2012). The use of metabolomics to dissect plant responses to abiotic stresses. Cell. Mol. Life Sci..

[23] Lesellier E., West C. (2015). The many faces of packed column supercritical fluid chromatography—A critical review. J. Chromatogr. A.

[24] John P.M., van D., Doris M.J. (2016). Assessment of dietary exposure and effect in humans: The role of NMR. Prog. Nucl. Magn. Reson. Spectrosc..

[25] Nagana Gowda G.A., Daniel R. (2015). Can NMR Solve Some Significant Challenges in Metabolomics. J. Magn. Reson..

[26] Imai A., Lankin D.C., Nikolić D., Ahn S., van Breemen R.B., Farnsworth N.R., McAlpine J.B., Chen S.N. (2016). Pauli GFCycloartane Triterpenes from the Aerial Parts of *Actaea racemosa*. J. Nat. Prod..

[27] Muhit M.A., Umehara K., Mori-Yasumoto K., Noguchi H. (2016). Furofuran Lignan Glucosides with Estrogen-Inhibitory Properties from the Bangladeshi Medicinal Plant *Terminalia citrina*. J. Nat. Prod..

[28] Chen Y., Xu J., Zhang R., Abliz Z. (2016). Methods used to increase the comprehensive coverage of urinary and plasma metabolomes by MS. Bioanalysis.

[29] Kueger S., Steinhauser D., Willmitzer L., Giavalisco P. (2012). High-resolution plant metabolomics: From mass spectral features to metabolites and from whole-cell analysis to subcellular metabolite distributions. Plant J..

[30] Jorge T.F., Mata A.T., António C. (2016). Mass spectrometry as a quantitative tool in plant metabolomics. Philos. Trans. A Math. Phys. Eng. Sci..

[31] Roessner U., Wagner C., Kopka J., Trethewey R.N., Willmitzer L. (2000). Technical advance: Simultaneous analysis of metabolites in potato tuber by gas chromatography-mass spectrometry. Plant J..

[32] Tsugawa H., Bamba T., Shinohara M., Nishiumi S., Yoshida M., Fukusaki E. (2011). Practical non-targeted gas chromatography/mass spectrometry-based metabolomics platform for metabolic phenotype analysis. J. Biosci. Bioeng..

[33] Kumari S., Stevens D., Kind T., Denkert C., Fiehn O. (2011). Applying in silico retention index and mass spectra matching for identification of unknown metabolites in accurate mass GC-TOF mass spectrometry. Anal. Chem..

[34] Kind T., Wohlgemuth G., Lee do Y., Lu Y., Palazoglu M., Shahbaz S., Fiehn O. (2009). FiehnLib: Mass spectral and retention index libraries for metabolomics based on quadrupole and time-of-light gas chromatography/mass spectrometry. Anal. Chem..

[35] Kopka J., Schauer N., Krueger S., Birkemeyer C., Usadel B., Bergmüller E., Dörmann P., Weckwerth W., Gibon Y., Stitt M. (2005). GMD@CSB.DB: The Golm Metabolome Database. Bioinformatics.

[36] Burgos A., Szymanski J., Seiwert B., Degenkolbe T., Hannah M.A., Giavalisco P., Willmitzer L. (2011). Analysis of short-term changes in the *Arabidopsis thaliana* glycerolipidome in response to temperature and light. Plant J..

[37] Nordström A., Want E., Northen T., Lehtio J., Siuzdak G. (2008). Multiple ionization mass spectrometry strategy used to reveal the complexity of metabolomics. Anal. Chem..

[38] An Z., Chen Y., Zhang R., Song Y., Sun J., He J., Bai J., Dong L., Zhan Q., Abliz Z. (2010). Integrated ionization approach for RRLC-MS/MS-based metabonomics: Finding potential biomarkers for lung cancer. J. Proteome Res..

[39] Tian H., Bai J., An Z., Chen Y., Zhang R., He J., Bi X., Song Y., Abliz Z. (2013). Plasma metabolome analysis by integrated ionization rapid-resolution liquid chromatography/tandem mass spectrometry. Rapid Commun. Mass Spectrom..

[40] Shao Y., Zhu B., Zheng R., Zhao X., Yin P., Lu X., Jiao B., Xu G., Yao Z. (2015). Development of urinary pseudotargeted LC-MS-based metabolomics method and its application in hepatocellular carcinoma biomarker discovery. J. Proteome Res..

[41] Lacorte S., Fernandez-Alba A.R. (2006). Time of flight mass spectrometry applied to the liquid chromatographic analysis of pesticides in water and food. Mass Spectrom. Rev..

[42] Mueller S.P., Krause D.M., Mueller M.J., Fekete A. (2015). Accumulation of extra-chloroplastic triacylglycerols in *Arabidopsis* seedlings during heat acclimation. J. Exp. Bot..

[43] Tarazona P., Feussner K., Feussner I. (2015). An enhanced plant lipidomics method based on multiplexed liquid chromatography-mass spectrometry reveals additional insights into cold- and drought-induced membrane remodeling. Plant J..

[44] Nakabayashi R., Mori T., Saito K. (2014). Alternation of flavonoid accumulation under drought stress in *Arabidopsis thaliana*. Plant Signal. Behav..

[45] Pires M.V., Pereira Júnior A.A., Medeiros D.B., Daloso D.M., Pham P.A., Barros K.A., Engqvist M.K., Florian A., Krahnert I., Maurino V.G. (2016). The influence of alternative pathways of respiration that utilize branched-chain amino acids following water shortage in *Arabidopsis*. Plant Cell Environ..

[46] Mao G., Seebeck T., Schrenker D., Yu O. (2013). CYP709B3, a cytochrome P450 monooxygenase gene involved in salt tolerance in *Arabidopsis thaliana*. BMC Plant Biol..

[47] Gigon A., Matos A.R., Laffray D., Zuily-Fodil Y., Pham-Thi A.T. (2004). Effect of drought stress on lipid metabolism in the leaves of *Arabidopsis thaliana* (ecotype Columbia). Ann. Bot..

[48] Gasulla F., Vom Dorp K., Dombrink I., Zähringer U., Gisch N., Dörmann P., Bartels D. (2013). The role of lipid metabolism in the acquisition of desiccation tolerance in *Craterostigma plantagineum*: A comparative approach. Plant J..

[49] Zarza X., Atanasov K.E., Marco F., Arbona V., Carrasco P., Kopka J., Fotopoulos V., Munnik T., Gómez-Cadenas A., Tiburcio A.F. (2016). Polyamine Oxidase 5 loss-of-function mutations in *Arabidopsis thaliana* trigger metabolic and transcriptional reprogramming and promote salt stress tolerance. Plant Cell Environ..

[50] Chen Y., Hoehenwarter W. (2015). Changes in the Phosphoproteome and Metabolome Link Early Signaling Events to Rearrangement of Photosynthesis and Central Metabolism in Salinity and Oxidative Stress Response in *Arabidopsis*. Plant Physiol..

[51] Guy C. (1999). Molecular responses of plants to cold shock and cold acclimation. J. Mol. Microbiol. Biotechnol..

[52] Nair P., Kandasamy S., Zhang J., Ji X., Kirby C., Benkel B., Hodges M.D., Critchley A.T., Hiltz D., Prithiviraj B. (2012). Transcriptional and metabolomic analysis of *Ascophyllum nodosum* mediated freezing tolerance in *Arabidopsis thaliana*. BMC Genom..

[53] Browse J., Lange B.M. (2004). Counting the cost of a cold-blooded life: Metabolomics of cold acclimation. Proc. Natl. Acad. Sci. USA.

[54] Moellering E.R., Benning C. (2011). Galactoglycerolipid metabolism under stress: A time for remodeling. Trends Plant Sci..

[55] Mittler R. (2002). Oxidative stress, antioxidants and stress tolerance. Trends Plant Sci..

[56] Chakraborty A., Bhattacharjee S. (2015). Differential competence of redox-regulatory mechanism under extremes of temperature determines growth performances and cross tolerance in two indica rice cultivars. J. Plant Physiol..

[57] Lobell D.B., Schlenker W. (2011). Costa-Roberts J. Climate trends and global crop production since 1980. Science.

[58] Li X.M., Chao D.Y., Wu Y., Huang X., Chen K., Cui L.G., Su L., Ye W.W., Chen H., Chen H.C. (2015). Natural alleles of a proteasome α2 subunit gene contribute to thermotolerance and adaptation of African rice. Nat. Genet..

[59] Battisti D.S., Naylor R.L. (2009). Historical warnings of future food insecurity with unprecedented seasonal heat. Science.

[60] Semenov M.A., Shewry P.R. (2011). Modelling predicts that heat stress, not drought, will increase vulnerability of wheat in Europe. Sci. Rep..

[61] Guy C., Kaplan F., Kopka J., Selbig J., Hincha D.K. (2008). Metabolomics of temperature stress. Physiol. Plant..

[62] Shulaev V., Cortes D., Miller G., Mittler R. (2008). Metabolomics for plant stress response. Physiol. Plant..

[63] Moellering E.R., Muthan B., Benning C. (2010). Freezing tolerance in plants requires lipid remodeling at the outer chloroplast membrane. Science.

[64] Schmelz E.A., Engelberth J., Alborn H.T., O’Donnell P., Sammons M., Toshima H., Tumlinson J.H. (2003). Simultaneous analysis of phytohormones, phytotoxins, and volatile organic compounds in plants. Proc. Natl. Acad. Sci. USA.

[65] Métraux J.P., Signer H., Ryals J., Ward E., Wyss-Benz M., Gaudin J., Raschdorf K., Schmid E., Blum W., Inverardi B. (1990). Increase in salicylic acid at the onset of systemic acquired resistance in cucumber. Science.

[66] Heil M., Bostock R.M. (2002). Induced systemic resistance (ISR) against pathogens in the context of induced plant defences. Ann. Bot..

[67] Ali M.A., Plattner S., Radakovic Z., Wieczorek K., Elashry A., Grundler F.M., Ammelburg M., Siddique S., Bohlmann H. (2013). An *Arabidopsis* ATPase gene involved in nematode-induced syncytium development and abiotic stress responses. Plant J..

[68] Matić S., Bagnaresi P., Biselli C., Orru’ L., Amaral Carneiro G., Siciliano I., Valé G., Gullino M.L., Spadaro D. (2016). Comparative transcriptome profiling of resistant and susceptible rice genotypes in response to the seedborne pathogen *Fusarium fujikuroi*. BMC Genom..

[69] Moore B., Zhou L., Rolland F., Hall Q., Cheng W.H., Liu Y.X., Hwang I., Jones T., Sheen J. (2003). Role of the *Arabidopsis* glucose sensor HXK1 in nutrient, light, and hormonal signaling. Science.

[70] Rolland F., Moore B., Sheen J. (2002). Sugar sensing and signaling in plants. Plant Cell.

[71] Vaclavik L., Mishra A., Mishra K.B., Hajslova J. (2013). Mass spectrometry-based metabolomic fingerprinting for screening cold tolerance in *Arabidopsis thaliana* accessions. Anal. Bioanal. Chem..

[72] Dixon R.A. (2001). Natural products and plant disease resistance. Nature.

[73] Espinoza C., Degenkolbe T., Caldana C., Zuther E., Leisse A., Willmitzer L., Hincha D.K., Hannah M.A. (2010). Interaction with diurnal and circadian regulation results in dynamic metabolic and transcriptional changes during cold acclimation in *Arabidopsis*. PLoS ONE.

[74] Tasseva G., de Virville J.D., Cantrel C., Moreau F., Zachowski A. (2004). Changes in the endoplasmic reticulum lipid properties in response to low temperature in *Brassica napus*. Plant Physiol. Biochem..

[75] Yu B., Benning C. (2003). Anionic lipids are required for chloroplast structure and function in *Arabidopsis*. Plant J..

[76] Chen M., Thelen J.J. (2013). *ACYL-LIPID DESATURASE2* is required for chilling and freezing tolerance in *Arabidopsis*. Plant Cell.

[77] Uemura M., Joseph R.A., Steponkus P.L. (1995). Cold acclimation of *Arabidopsis thaliana* (effect on plasma membrane lipid composition and freeze-induced lesions). Plant Physiol..

[78] Welti R., Li W., Li M., Sang Y., Biesiada H., Zhou H.E., Rajashekar C.B., Williams T.D., Wang X. (2002). Profiling membrane lipids in plant stress responses. Role of phospholipase Dα in freezing-induced lipid changes in *Arabidopsis*. J. Biol. Chem..

[79] Li W., Wang R., Li M., Li L., Wang C., Welti R., Wang X. (2008). Differential degradation of extraplastidic and plastidic lipids during freezing and post-freezing recovery in *Arabidopsis thaliana*. J. Biol. Chem..

[80] Routaboul J.M., Fischer S.F., Browse J. (2000). Trienoic fatty acids are required to maintain chloroplast function at low temperatures. Plant Physiol..

[81] Koffler B.E., Luschin-Ebengreuth N., Stabentheiner E., Müller M., Zechmann B. (2014). Compartment specific response of antioxidants to drought stress in *Arabidopsis*. Plant Sci..

[82] Ribas-Carbo M., Taylor N.L., Giles L., Busquets S., Finnegan P.M., Day D.A., Lambers H., Medrano H., Berry J.A., Flexas J. (2005). Effects of water stress on respiration in soybean leaves. Plant Physiol..

[83] Skirycz A., de Bodt S., Obata T., de Clercq I., Claeys H., de Rycke R., Andriankaja M., van Aken O., van Breusegem F., Fernie A.R. (2010). Developmental stage specificity and the role of mitochondrial metabolism in the response of *Arabidopsis* leaves to prolonged mild osmotic stress. Plant Physiol..

[84] Gilbert N. (2012). Drought devastates US crops. Nature.

[85] Varshney R.K., Ribaut J.M., Buckler E.S., Tuberosa R., Rafalski J.A., Langridge P. (2012). Can genomics boost productivity of orphan crops?. Nat. Biotechnol..

[86] Saito K., Yonekura-Sakakibara K., Nakabayashi R., Higashi Y., Yamazaki M., Tohge T., Fernie A.R. (2013). The flavonoid biosynthetic pathway in *Arabidopsis*: Structural and genetic diversity. Plant Physiol. Biochem..

[87] Dixon R.A., Paiva N.L. (1995). Stress-Induced Phenylpropanoid Metabolism. Plant Cell.

[88] Rowan D.D., Cao M., Lin-Wang K., Cooney J.M., Jensen D.J., Austin P.T., Hunt M.B., Norling C., Hellens R.P., Schaffer R.J. (2009). Environmental regulation of leaf colour in red 35S: PAP1 *Arabidopsis thaliana*. New Phytol..

[89] Stracke R., Favory J.J., Gruber H., Bartelniewoehner L., Bartels S., Binkert M., Funk M., Weisshaar B., Ulm R. (2010). The *Arabidopsis* bZIP transcription factor HY5 regulates expression of the *PFG1/MYB12* gene in response to light and ultraviolet-B radiation. Plant Cell Environ..

[90] Catala R., Medina J., Salinas J. (2011). Integration of low temperature and light signaling during cold acclimation response in *Arabidopsis*. Proc. Natl. Acad. Sci. USA.

[91] Koops P., Pelser S., Ignatz M., Klose C., Marrocco-Selden K., Kretsch T. (2011). EDL3 is an F-box protein involved in the regulation of abscisic acid signalling in *Arabidopsis thaliana*. J. Exp. Bot..

[92] Kusano M., Tohge T., Fukushima A., Kobayashi M., Hayashi N., Otsuki H., Kondou Y., Goto H., Kawashima M., Matsuda F. (2011). Metabolomics reveals comprehensive reprogramming involving two independent metabolic responses of *Arabidopsis* to UV-B light. Plant J..

[93] Lei M.G., Liu Y.D., Zhang B.C., Zhao Y.T., Wang X.J., Zhou Y.H., Raghothama K.G., Liu D. (2011). Genetic and genomic evidence that sucrose is a global regulator of plant responses to phosphate starvation in *Arabidopsis*. Plant Physiol..

[94] Tohge T., Watanabe M., Hoefgen R., Fernie A.R. (2013). The evolution of phenylpropanoid metabolism in the green lineage. Crit. Rev. Biochem. Mol. Biol..

[95] Nakabayashi R., Yonekura-Sakakibara K., Urano K., Suzuki M., Yamada Y., Nishizawa T., Matsuda F., Kojima M., Sakakibara H., Shinozaki K. (2014). Enhancement of oxidative and drought tolerance in *Arabidopsis* by overaccumulation of antioxidant flavonoids. Plant J..

[96] Bari R., Jones J.D. (2009). Role of plant hormones in plant defence responses. Plant Mol. Biol..

[97] Rai A., Umashankar S., Rai M., Kiat L.B., Bing J.A., Swarup S. (2016). Coordinate Regulation of Metabolite Glycosylation and Stress Hormone Biosynthesis by TT8 in *Arabidopsis*. Plant Physiol..

[98] Seo P.J., Lee A.K., Xiang F., Park C.M. (2008). Molecular and functional profiling of *Arabidopsis* pathogenesis-related genes: Insights into their roles in salt response of seed germination. Plant Cell Physiol..

[99] Liu W.X., Zhang F.C., Zhang W.Z., Song L.F., Wu W.H., Chen Y.F. (2013). *Arabidopsis* Di19 functions as a transcription factor and modulates *PR1*, *PR2*, and *PR5* expression in response to drought stress. Mol. Plant.

[100] Mir R., Hernández M.L., Abou-Mansour E., Martínez-Rivas J.M., Mauch F., Métraux J.P., León J. (2013). Pathogen and Circadian Controlled 1 (PCC1) regulates polar lipid content, ABA-related responses, and pathogen defence in *Arabidopsis thaliana*. J. Exp. Bot..

[101] Caldana C., Degenkolbe T., Cuadros-Inostroza A., Klie S., Sulpice R., Leisse A., Steinhauser D., Fernie A.R., Willmitzer L., Hannah M.A. (2011). High-density kinetic analysis of the metabolomic and transcriptomic response of *Arabidopsis* to eight environmental conditions. Plant J..

[102] Hildebrandt T.M., Nunes Nesi A., Araújo W.L. (2015). Braun HP4. Amino Acid Catabolism in Plants. Mol. Plant.

[103] Paul M.H. (2013). Sodium (Na^+^) homeostasis and salt tolerance of plants. Environ. Exp. Bot..

[104] Munns R., Tester M. (2008). Mechanisms of salinity tolerance. Annu. Rev. Plant Biol..

[105] Bednarek P., Pislewska-Bednarek M., Svatos A., Schneider B., Doubsky J., Mansurova M., Humphry M., Consonni C., Panstruga R., Sanchez-Vallet A. (2009). A glucosinolate metabolism pathway in living plant cells mediates broad-spectrum antifungal defense. Science.

[106] Oliver M.J., Guo L., Alexander D.C., Ryals J.A., Wone B.W., Cushman J.C. (2011). A sister group contrast using untargeted global metabolomic analysis delineates the biochemical regulation underlying desiccation tolerance in *Sporobolus stapfianus*. Plant Cell.

[107] Burg M.B., Ferraris J.D. (2008). Intracellular organic osmolytes: Function and regulation. J. Biol. Chem..

[108] Kol S., Merlo M.E., Scheltema R.A., de Vries M., Vonk R.J., Kikkert N.A., Dijkhuizen L., Breitling R., Takano E. (2010). Metabolomic characterization of the salt stress response in *Streptomyces coelicolor*. Appl. Environ. Microbiol..

[109] Liu J., Zhu J.K. (1997). Proline accumulation and salt-stress-induced gene expression in a salt-hypersensitive mutant of *Arabidopsis*. Plant Physiol..

[110] Strizhov N., Abrahám E., Okrész L., Blickling S., Zilberstein A., Schell J., Koncz C., Szabados L. (1997). Differential expression of two *P5CS* genes controlling proline accumulation during salt-stress requires ABA and is regulated by *ABA1*, *ABI1* and *AXR2* in *Arabidopsis*. Plant J..

[111] Verslues P.E., Sharma S. (2010). Proline metabolism and its implications for plant-environment interaction. Arabidopsis Book.

[112] Yamaguchi K., Takahashi Y., Berberich T., Imai A., Miyazaki A., Takahashi T., Michael A., Kusano T. (2006). The polyamine spermine protects against high salt stress in *Arabidopsis thaliana*. FEBS Lett..

[113] Ashraf M., Harris P.J.C. (2004). Potential biochemical indicators of salinity tolerance in plants. Plant Sci..

[114] Wu W., Zhang Q., Zhu Y., Lam H.M., Cai Z., Guo D. (2008). Compartive metabolic profiling reveals secondary metabolites correlated with soybean salt tolerance. J. Agric. Food Chem..

[115] Yeo A. (1998). Molecular biology of salt tolerance in the context of whole-plant physiology. J. Exp. Bot..

[116] Liu Y.F., Li Q.T., Lu X., Song Q.X., Lam S.M., Zhang W.K., Ma B., Lin Q., Man W.Q., Du W.G. (2014). Soybean *GmMYB73* promotes lipid accumulation in transgenic plants. BMC Plant Biol..

[117] Cheong W.F., Wenk M.R., Shui G. (2014). Comprehensive analysis of lipid composition in crude palm oil using multiple lipidomic approaches. J. Genet. Genom..

